# Separation of Volatile Metabolites from the Leaf-Derived Essential Oil of *Piper mollicomum* Kunth (Piperaceae) by High-Speed Countercurrent Chromatography

**DOI:** 10.3390/molecules23123064

**Published:** 2018-11-23

**Authors:** André M. Marques, Ana Clarissa C. Peixoto, D. William Provance, Maria Auxiliadora C. Kaplan

**Affiliations:** 1 Laboratório de Produtos Naturais, PN3, FarManguinhos, FIOCRUZ, 21045-900 Rio de Janeiro, Brazil; 2 Instituto de Pesquisas de Produtos Naturais (IPPN), Universidade Federal do Rio de Janeiro (UFRJ), 21941-902 Rio de Janeiro, Brazil; acpeixoto1@ufrj.br (A.C.C.P.); kaplan@nppn.ufrj.br (M.A.C.K.); 3 Centro de Desenvolvimento Tecnológico em Saúde (CDTS/Fiocruz), 21045-360 Rio de Janeiro, Brasil; bill.provance@cdts.fiocruz.br

**Keywords:** camphor, nerolidol, separation process, CCC, essential oil

## Abstract

The technique of high-speed countercurrent chromatography was applied to the isolation of compounds in essential oil derived from the leaves of *Piper mollicomum* species. Plant leaves (200.0 g) were submitted to hydrodistillation in a modified Clevenger apparatus. The resulting crude leaf essential oil was analyzed by gas chromatography with flame ionization detector (GC-FID) and gas chromatography-mass spectrometry (GC-MS) to determine the profile of the components. The purified fractions were composed of monoterpenes and sesquiterpenes such as camphor (85.0 mg at 98.5% purity), (*E*)-nerolidol (100.0 mg at 92.8% purity), and camphene (150.0 mg at 82.0% purity). A minor component of the essential oil, bornyl acetate (16.2 mg at 91.2% purity) was also isolated in the one-step separation protocol in 2 h. The countercurrent chromatography technique proved to be a fast and efficient method for the separation of volatile metabolites that conserved the solvent while delivering various fractions of high purity.

## 1. Introduction

Natural product drug discovery can be an expensive and time-consuming process to isolate active constituents [1,2,3]. The isolation of pure bioactive compounds from natural sources on a preparative or semi-preparative scale is frequently necessary for further pharmacological investigations [4]. Yet, the isolation of natural compounds is normally a complex process requiring a comprehensive range of separation techniques that often present multiple challenges including irreversible adsorption, low yield, and low purity. A frequent source of natural bioactive compounds are essential oils derived from plants, which are hydrophobic liquids containing a variety of volatile aromatic compounds. The chemical composition of essential oils is generally very complex and difficult to separate in high yields of sufficient purity [5,6]. The essential oil of *Piper mollicomum* has an economic potential due to presence of camphor, camphor derivates, and nerolidol, which are expensive compounds of which separation through more economical methods would be of considerable interest to the pharmaceutical and perfume industries [7,8,9].

Some of the main constituents of essential oils are monoterpenes and sesquiterpenes that represent a large and diverse group of natural products. They share a number of important properties. They display a wide range of biological activities including antibacterial [10], antiviral [11], antioxidant [12], and antiparasitic activity [13]. They also have similarities in structure, volatility, weakness in polarity, and a lack of UV absorption that create a number of challenges for their preparative separation by conventional methods based on chromatographic columns [14]. Frequently, column chromatography based on silica gel is used to separate volatile terpenes. However, it is time-consuming and, in most cases, the major components in essential oils cannot be retained on silica gel [15]. Other complications arise from the use of a silica stationary phase in liquid chromatography such as irreversible solute adsorption, contamination, size exclusion, residual silanols, and pH limitations that affect yield and purity [16]. Advances have been achieved in the isolation of terpenes by vacuum liquid chromatography and high-pressure liquid chromatography, which are semi-preparative liquid–solids techniques [17,18]. However, the use of liquid–liquid isolation methods, such as high-speed countercurrent chromatography (HSCCC), improved the separation of secondary metabolism compounds due to its greater selectivity [19,20].

Countercurrent chromatography (CCC) is a modern chromatographic separation technology that has shown significant potential to contribute to new approaches in natural product drug discovery, specifically towards reducing the expensive and time-consuming steps to isolate active constituents [21]. This liquid–liquid chromatographic method permits both normal and reversed-phase separation procedures that are useful in the isolation process of compounds that normally have limited thermal and chemical stability, such as volatile components in essential oils [22]. As a support-free liquid–liquid partition chromatographic technique, it eliminates the potential for the irreversible adsorption of samples onto the solid support of traditional techniques [23,24,25]. Furthermore, recent advances in CCC equipment provide an additional advantage from the increased sample loading capacity for performing preparative-scale separations [23].

Here, we report on the development of a chromatography method based on HSCCC for the isolation of the compounds from the essential oil obtained from the leaves of *P. mollicomum*. The limited complexity and similarities between different components of this essential oil permitted a comprehensive evaluation of performance. Based on the gas chromatography mass spectrometry analyses of the beginning material and resulting purified fractions, HSCCC proved to be a rapid and effective technique for fractionating the volatile components to high purity at milligram quantities.

## 2. Results and Discussion

**Figure 1:**
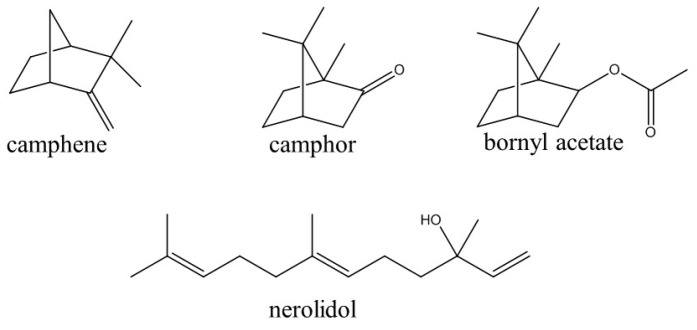
Chemical structures of volatile compounds isolated from the leaf essential oil of *Piper mollicomum*.

In this work, HSCCC was applied for a fast and economic separation of the constituents in essential oil derived from *P. mollicomum* leaves. Using this technique, the level of solvent consumption was comparatively much less than those using techniques based on conventional chromatographic columns [26]. In this process, the HSCCC technique was shown as a useful tool in the separation of the volatile compounds that normally have limited thermal and chemical stability [24]. The essential oils obtained from the fresh leaves of *P. mollicomum* were first analyzed by GC-FID and GC-MS. The leaf essential oil was characterized by its high percentage of camphor (39.9 ± 1.9%), camphene (25.3 ± 2.1%), and nerolidol (7.5 ± 1.1%) that also had a minor component of bornyl acetate (2.4 ± 0.2%) in the composition, as shown in Figure 1. In total, fifteen components were identified in this essential oil by GC-MS that encompassed 98.0% of the entire composition, as shown in Table 1.

The execution of a successful separation process using countercurrent chromatography requires a careful search for a suitable two-phase solvent system that can provide an ideal range of partition coefficients (K) for the target components. Since most volatile terpenoids have strong hydrophobic properties, the most commonly described HSCCC solvent systems in the literature for this propose are composed of: *n*-hexane/ACN, *n*-hexane/AcoEt/ACN, *n*-hexane/ACN/MeOH, petroleum ether/ethyl acetate/methanol/water, petroleum ether/ethanol/water, and *n*-hexane/ACN/chloroform [22]. An ideal partition coefficient for a targeted compound generally ranges from 0.5–2.0 for a specific solvent system. A large partition coefficient generally leads to longer retention times while smaller partition coefficients can generally result in poor resolution of the compounds peak. Another consideration is that the two-phase solvent system should display high solubility and stability for target compounds along with the physical characteristics of a short settling time (<30 s) and a satisfactory stationary phase retention of greater than 50% [21]. In this study, five combinations of two-phase solvent systems were tested together. Their measured K values are presented in Table 2.

The solvent systems *n*-hexane-methanol (1:1, *v*/*v*), *n*-hexane-acetonitrile (1:1, *v*/*v*), petroleum ether/acetonitrile/acetone (4:3:1, *v*/*v*/*v*), n-hexane/acetonitrile/ethyl acetate (1:1:0.4, *v*/*v*/*v*), and n-hexane/acetonitrile/methanol (1:1:0.5, *v*/*v*/*v*) were investigated for separation of components from essential oil by countercurrent chromatography. The hydrophobic solvent systems composed of acetonitrile were considered the best choice in the usual non-aqueous two-phase solvent systems for a number of reasons.

Based on the calculated K values for the target compounds, the systems containing methanol were not considered the most suitable for use as solvent systems. The K value for nerolidol of >2 would lead to a long run and the K value for camphene of <0.1 would most likely prove too short of a time in the column to efficiently separate this compound in the partitioning process. In addition, the solvent systems 2 and 4 containing MeOH were also not considered utile for this separation since they presented lower volatility in comparison to the systems 1, 3, and 5, which could limit the mass recovery of compounds. Recovery of volatile compounds by evaporation of HSCCC fractions would be complicated if either methanol or water were contained in the two-phase solvent system. Lastly, in consideration of solubility, the major volatile terpenes are not soluble to the same extent in polar protonic solvents like methanol as in systems containing *n*-hexane-acetonitrile, which could limit the capacity and yield of the system.

Compared to systems 1 and 3 that also contain acetonitrile, system 5, with the presence of petrol, presented very similar distribution coefficient (KD) values for the target compounds camphor and bornyl acetate. The calculated KD values for camphene, camphor, and bornyl acetate in system 5 of 0.09, 0.87, and 0.70 respectively were all lower than 1.0, which could lead to a decreased resolution of the separation process. Thus, these compounds would be eluted easily by the mobile phase, having a short time to be properly separated. Based on K values for the target compounds, the best separation results were achieved by using *n*-hexane/acetonitrile (1:1) and *n*-hexane/acetonitrile/ethyl acetate (1:1:0.4) as solvent systems.

The two-phase solvent systems containing *n*-hexane/acetonitrile were tested in HSCCC running for *P. mollicomum* essential oil separation. Preliminary tests showed that by using system 1 (Hex/ACN, 1:1) in the HSCCC separation process, it was not possible to separate and obtain the 4 target compounds in a single one-step process with a high purity level, especially for camphene and bornyl acetate, which were easily eluted by the mobile phase together with many terpenes (data not shown). Although both systems are considered suitable for use in HSCCC separation, system 3 was chosen to be used in the separation process based on the considerable difference between their KD values. The insertion of ethyl acetate to system 1 (Hex/ACN) increased the partition coefficient of camphene from 0.07 to 0.37, camphor from 1.13 to 1.40, and bornyl acetate from 0.85 to 1.20. This improved the efficiency and resolution of this separation process by elevating the partition of these metabolites between both solvent phases. Under the optimal condition, an isocratic elution was conducted for the separation of the crude essential oil (1.4 g) using *n*-hexane/acetonitrile/ethyl acetate (1:1:0.4) separation in a one-step process over a 120 min run time in a tail-to-head elution mode. The flow rate of the mobile phase was selected as 2.0 mL/min. Nearly 73 mL (91.2%) of the stationary phase was retained after the hydrodynamic equilibrium was attained in this process.

**Figure 2:**
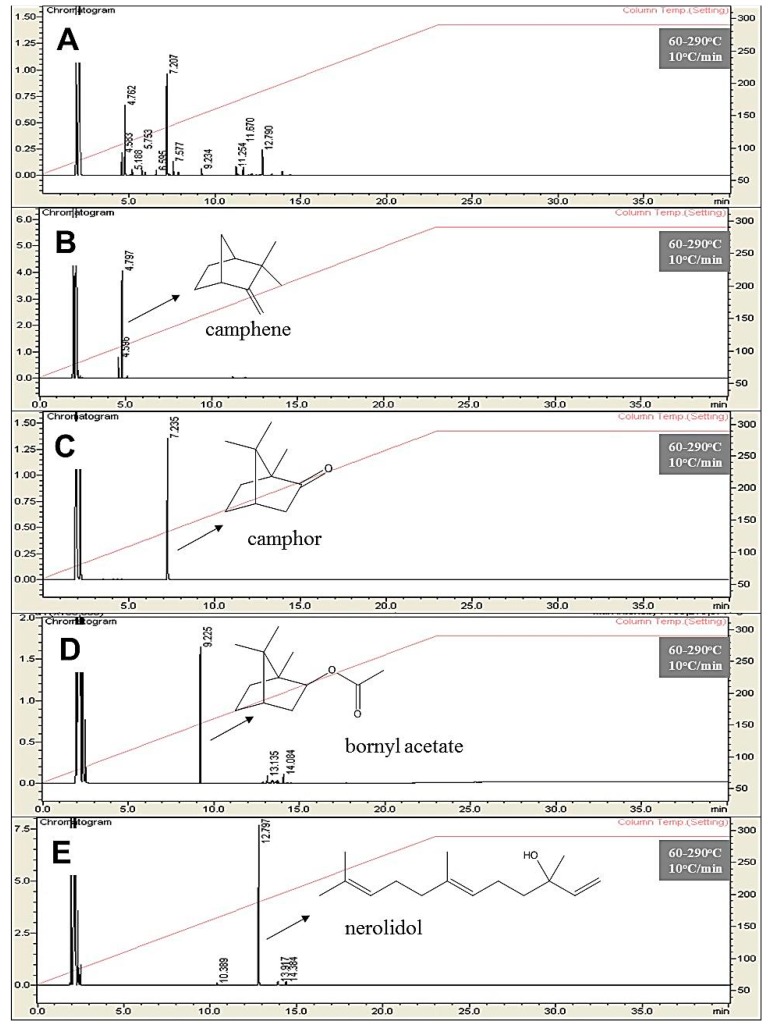
GC-FID chromatograms of *P. mollicomum* leaf essential oil and high-speed countercurrent chromatography (HSCCC) metabolites separation. (**A**) Crude *P. mollicomum* leaf essential oil. (**B**) Camphene HSCCC fraction. (**C**) Camphor HSCCC fraction. (**D**) Bornyl acetate HSCCC Fraction. (**E**) Nerolidol HSCCC fraction.

As shown in Figure 2, the developed preparative HSCCC method was successfully used for the separation of the major metabolites from *P. mollicomum* essential oil. In the one-step separation process (2 h), it was possible to obtain 150.0 mg of camphene (1), 85.0 mg of camphor (2), 16.2 mg of bornyl acetate (3), and 100.0 mg of (*E*)-nerolidol (4). The purities of the four fractions isolated by this HSCCC system were analyzed by GC-FID, whose results revealed camphene (82.0%), camphor (98.5%), bornyl acetate (91.2%), and (*E*)-nerolidol (92.8%). The structural identification of the HSCCC peak fractions was obtained by ^1^H-NMR and ^13^C-NMR. Thus, the peak fractions 1–4 were characterized and identified as camphene (1), camphor (2), bornyl acetate (3), and (*E*)-nerolidol (4) respectively.

## 3. Materials and Methods

### 3.1. Solvents and Reagents

All of the organic solvents used for HSCCC were of analytical grade.

### 3.2. Essential Oil Extraction

Leaves (200.0 g) of *P. mollicomum* Kunth were collected on the campus of the Federal University of Rio de Janeiro, Ilha do Fundão, Rio de Janeiro in June 2017. The botanical vouchers were identified and kept at the herbarium of the Botanical Garden of Rio de Janeiro (Register number RB 606975). Fresh leaf material of the plant was submitted to hydrodistillation for 2 h in a modified Clevenger-type apparatus. The obtained essential oil was dried over anhydrous sodium sulphate, yielding 0.9% (*w*/*w*) in leaf essential oil samples.

### 3.3. Preparation of the Two-Phase Solvent System and Sample Solutions

For HSCCC, five different solvent systems were tested with each system generating a two-phase mixture when mixed at the stated volume to volume ratio: System 1—*n*-hexane and methanol (1:1); system 2—*n*-hexane and acetonitrile (1:1); system 3—petroleum ether, acetonitrile, and acetone (4:3:1); system 4—*n*-hexane, acetonitrile, and ethyl acetate (1:1:0.4); and system 5—*n*-hexane, acetonitrile, and methanol (1:1:0.5). The solutions containing samples were prepared by diluting essential oil into a mixed solution of the lower phase and upper phase (1:1, *v*/*v*) of the solvent system for HSCCC separation. The solution was vigorously shaken and the two phases allowed to settle. The distribution of the essential oil components into the upper and lower phases was first estimated by thin-layer chromatography (TLC, silica gel 60 F254 nm) of equal volume samples with Hexane/Ethyl acetate (3:2) as the eluting solvent system. The final separation of compounds was observed under a UV lamp at 254 nm. To assist the visual estimation of the relative distribution of the compounds in each phase, a sulphuric acid/methanol reagent (1:1, *v*/*v*) was sprayed onto the sample followed by heating. The KDs were calculated by GC-FID analyses.

### 3.4. Determination of Distribution Coefficient for the Major Terpene Compounds by GC-FID

One drop (approximately 5 mg) of the leaf essential oil from *P. mollicomum* was added to the mixture of equal volumes of the upper phase and the lower phase from the two-phase solvent system in each flask (Section 3.3) and each phase analyzed separately by GC-FID using the conditions described in Section 3.6. The solvent systems were composed of 1 mL of both phases in each flask. The KD was calculated as KD = SP (peak area of compound)/MP (peak area of compound), where SP and MP represent the GC-FID peak areas of *P. mollicomum* essential oil components in the stationary and mobile phases, respectively. The calculated KD values are shown in Table 2.

### 3.5. Apparatus and Separation Procedure

A CCC system (model HSCCC No. 403, PC Inc., Potomac, Montgomery, MD, USA), consisting of a PTFE 80-mL coil, an HPLC pump (model M-45, Waters, Milford, MA, USA), a low-pressure injection valve (Rheodyne 5020, Cotate, CA, USA), and a PTFE 5-mL sample loop was used. This system was coupled to a fraction collector (model L-7650, Merck, Darmstadt, Germany) programmed to collect at 1 min intervals. Appropriate volumes of the solvents *n*-hexane/acetonitrile/ethyl acetate (1:1:0.4) were vigorously hand-mixed in a separator funnel, transferred to a flask, and degassed (ultrasonic bath) for 30 min. An isocratic elution was conducted in a tail-to-head manner. The coil was entirely filled with the stationary phase of the solvent system with no rotation applied. The coil rotation was then initiated at 860 rpm and the upper organic phase was pumped at a flow rate of 2.0 mL/min. The system was allowed to attain the hydrodynamic equilibrium prior to the sample injection. The crude essential oil (1.4 g) was dissolved in the biphasic solvent systems and injected into the apparatus. Fractions (*n* = 120) of 2 mL each were collected in the span of 120 min. The first 80 fractions were collected with rotation “on” and the last 40 fractions with rotation “off”. All the fractions were monitored by TLC and the target fractions considered visually pure were analyzed by GC-FID and GC-MS. The fractions considered pure by CG-FID were analyzed by nuclear magnetic resonance (^1^H and ^13^C-NMR).

### 3.6. Gas Chromatography–Mass Spectrometry (GC–MS) Analysis

#### 3.6.1. GC-FID Analysis

The qualitative and quantitative analyses were carried out on a GC 2010 Shimadzu with a DB-5MS fused silica capillary column (30 m × 0.25 mm × 0.25 μm film thickness). The operating temperatures used were as follows: Injector 260 °C, detector 290 °C, and column oven 60 °C that increased to 290 °C by 10 °C/min. Hydrogen was used as a carrier gas at 1.0 mL min^−1^. The method was tested for repeatability by performing separations in triplicate. The yield percentages of a compound were determined by GC-FID analysis.

#### 3.6.2. GC-MS Analysis

Qualitative analyses were carried out on a GC-QP2010 PLUS Shimadzu with a ZB-5MS fused silica capillary column (30 m × 0.25 mm × 0.25 μm film thickness) under the experimental conditions reported for GC-FID analysis. The essential oil components were identified by comparing their retention indices and mass spectra to published data and computer matching with WILEY 275 and the National Institute of Standards and Technology (NIST 3.0) libraries provided by a computer-controlled GC-MS system. The results were also confirmed by comparing the compounds’ elution order with their relative retention indices reported in the literature [27]. The retention indices were calculated for all the volatile constituents using the retention data of linear *n*-alkanes C8–C24.

### 3.7. Nuclear Magnetic Resonance Spectroscopy

The isolated constituents obtained by the HSCCC separation were analyzed by ^1^H and ^13^C-NMR and recorded on a Brüker DRX 400 spectrometer. The chemical shifts were determined in CDCl3, using TMS as the internal standard. The signals of the NMR analyses were compared to the literature data [28,29].

## 4. Conclusions

Here, the components in essential oil from the leaves of *P. mollicomum* were separated in a one-step preparative separation process based on high-speed countercurrent chromatography. The technique showed high resolution for the separation of volatile metabolites that display very similar structures and polarity. High purity fractions were recovered over a short run time (<2 h) and the process proved to require less solvent than conventional techniques. An important aspect to this approach was the determination of an appropriate solvent system. Overall, high-speed countercurrent chromatography is a great selective tool for the separation of terpenes and, in particular, the complex compounds contained in botanical essential oils. The HSCCC can be used for the fast isolation of substances of commercial interest such as camphor and nerolidol.

## Figures and Tables

**Table 1:** Chemical composition and retention indices of the constituents of *P. mollicomum* leaf essential oil.

Compounds ^a^	RI		% (Average ± SD) ^c^	Identification
	Lit	Cal ^b^		
α-pinene	932	976	6.3 ± 0.3	RI, GC-MS
camphene	946	1004	25.3 ± 1.0	RI, GC-MS
β-pinene	974	1101	0.4 ± 0.8	RI, GC-MS
β-mircene	988	992	2.2 ± 0.1	RI, GC-MS
hepten-2-ol <6-methyl-5->	989	990	0.7 ± 0.1	RI, GC-MS
limonene	1024	1025	2.9 ± 0.4	RI, GC-MS
(*Z*)-β-ocimene	1032	1035	0.7 ± 0.1	RI, GC-MS
(*E*)-β-ocimene	1044	1049	1.2 ± 0.5	RI, GC-MS
camphor	1141	1145	39.9 ± 1.2	RI, GC-MS
borneol	1165	1166	1.3 ± 0.7	RI, GC-MS
Isobornyl acetate	1287	1290	2.4 ± 0.9	RI, GC-MS
α-caryophylene	1417	1419	3.7 ± 0.1	RI, GC-MS
(*Z*)-β-farnesene	1440	1445	2.9 ± 0.2	RI, GC-MS
α-amorphene	1511	1516	0.6 ± 0.1	RI, GC-MS
(*E)*-nerolidol	1561	1564	7.5 ± 0.5	RI, GC-MS
Total identified			98.0 ± 1.7	

^a^ Compounds are listed in order of their elution from the DB-5ms column. The RI-Lit values are obtained from reports in the literature [27]. ^b^ The RI-Cal values were experimentally determined against n-alkanes on the DB-5ms column. ^c^ Content is expressed as percentages obtained by integration of the GC peak area. Data are presented as average of three analyses performed in triplicate. Standard deviation (± SD).

**Table 2:** Distribution coefficient (*KD*) values in different solvent systems tested for the preparative isolation of terpenes from *P. mollicomum* leaf essential oil.

MODE	System 1	System 2	System 3	System 4	System 5
tail-to-head	Hex/CAN (1:1)	Hex/MeOH (1:1)	Hex/ACN/AcOEt (1:1:0.4)	Hex/ACN/MeOH (1:1:0.5)	Petrol/ACN/ACE (4:3:1)
camphene	0.07	0.09	0.37	0.07	0.09
camphor	1.13	1.40	1.47	1.55	0.87
bornyl acetate	0.85	1.20	0.73	1.47	0.70
nerolidol	1.71	3.75	2.06	2.92	2.35

## References

[1] Li G., Lou H.X. (2018). Strategies to diversify natural products for drug discovery. Med. Res. Rev..

[2] Cheuka P.M., Mayoka G., Mutai P., Chibale K. (2017). The Role of Natural Products in Drug Discovery and Development against Neglected Tropical Disease. Molecules.

[3] Shen B. (2015). A New Golden Age of Natural Products Drug Discovery. Cell.

[4] Carini J.P., Leitão G.G., Schneider P.H., Santos C.C., Costa F.N., Holzschuh M.H., Klamt F., Bassani V.L. (2015). Isolation of achyrobichalcone from Achyrocline satureioides by high- speed countercurrent chromatography. Curr. Pharm. Biotechnol..

[5] Ingle K.P., Deshmukh A.G., Padole D.A., Dudhare M.S., Moharil M.P., Khelurkar V.C. (2017). Phytochemicals: Extraction methods, identification and detection of bioactive compounds from plant extracts. J. Pharmacogn. Phytochem..

[6] Rassem H.H.A., Nour A.H., Yunus R.M. (2016). Techniques for Extraction of Essential Oils from Plants: A Review. Aust. J. Basic Appl. Sci..

[7] Santos P.R.D., Moreira D.L., Guimarães E.F., Kaplan M.A.C. (2001). Essential oil analysis of 10 Piperaceae species from the Brazilian Atlantic forest. Phytochemistry.

[8] Masetto M.A.M., Deschamps C., Mógor A.F., Bizzo H.R. (2011). Yield and composition of essential oil from inflorescences and leaves of lavender (*Lavandula dentata* L.) in different flower development stages and harvest times. Rev. Bras. Planta Med..

[9] Rezende K.R., Barros S.B.M. (2004). Quantification of 4-nerolidylcatechol from *Pothomorphe umbellate* (Piperaceae) in rat plasma samples by HPLC-UV. Braz. J. Pharm. Sci..

[10] Heydari M., Zanfardino A., Taleei A., Bushehri A.A.S., Hadian J., Maresca V., Sorbo S., Di Napoli M., Varcamonti M., Basile A. (2018). Effect of Heat Stress on Yield, Monoterpene Content and Antibacterial Activity of Essential Oils of *Mentha* x *piperita* var. *Mitcham* and *Mentha arvensis* var. *piperascens*. Molecules.

[11] Astani A., Reichling J., Schnitzler P. (2010). Comparative study on the antiviral activity of selected monoterpenes derived from essential oils. Phytother. Res..

[12] Amorati R., Foti M.C., Valgimigli L. (2013). Antioxidant Activity of essential Oils. J. Agric. Food Chem..

[13] Ogungbe I.F., Setzer W.N. (2013). In-silico Leishmania Target Selectivity of Antiparasitic Terpenoids. Molecules.

[14] Dhanarasu S. (2012). Chromatography and Its Applications.

[15] Jiang Z., Kempinski C., Chappell J. (2016). Extraction and Analysis of Terpenes/Terpenoids. Curr. Protoc. Plant. Biol..

[16] Berthod A., Ruiz-Ángel M.J., Carda-Broch S. (2009). Countercurrent chromatography: People and applications. J. Chromatogr. A.

[17] Coll J.C., Bowden B.F. (1986). The Application of Vacuum Liquid Chromatography to the Separation of Terpene Mixtures. J. Nat. Prod..

[18] Morin P., Caude M., Richard H., Rosset R. (1986). Semipreparative separation of terpenoids from essential oils by high-performance liquid chromatography and their subsequent identification by gas chromatography—Mass spectrometry. J. Chromatogr. A.

[19] Chen Q., Lin H., Wu X., Song H., Zhu X. (2018). Preparative separation of six terpenoids from *Wedelia prostrata* Hemsl. by two-step high-speed counter-current chromatography. J. Liq. Chromatogr. Relat. Technol..

[20] Sticher O. (2008). Natural product isolation. Nat. Prod. Rep..

[21] Pauli G.F., Pro S.M., Friesen J.B. (2008). Countercurrent separation of natural products. J. Nat. Prod..

[22] Woźniak K.S., Garrard I. (2014). Counter-current chromatography for the separation of terpenoids: A comprehensive review with respect to the solvent systems employed. Phytochem. Rev..

[23] Song H., Lin J., Zhu X., Chen Q. (2016). Developments in high-speed countercurrent chromatography and its applications in the separation of terpenoids and saponins. J. Sep. Sci..

[24] Michel T., Destandau T., Elfakir C. (2014). New advances in countercurrent chromatography and centrifugal partition chromatography: Focus on coupling strategy. Anal. Bioanal. Chem..

[25] Marques A.M., Fingolo C.E., Kaplan M.A.C. (2017). HSCCC separation and enantiomeric distribution of key volatile constituents of *Piper claussenianum* (Miq.) C. DC. (Piperaceae). Food Chem. Toxicol..

[26] Sutherland I.A. (2007). Recent progress on the industrial scale-up of counter-current chromatography. J. Chromatogr. A.

[27] Adams R.P. (2007). Identification of Essential Oil Components by Gas Chromatography/Quadrupole Mass Spectrometry.

[28] Marques A.M., Barreto A.L., Batista E.M., Curvelo J.A., Velozo L.S., Moreira D.L., Guimarães E.F., Soares R.M., Kaplan M.A. (2010). Chemistry and Biological Activity of Essential Oils from *Piper claussenianum* (Piperaceae). Nat. Prod. Commun..

[29] Yoneda J.D., Leal K.Z., Seidl P.R., Azeredo R.B.V., Kleinpeter E. (2007). Camphor: A good model for illustrating NMR techniques. Quim. Nova.

